# Determining the Role of OsAGP6P in Anther Development Within the Arabinogalactan Peptide Family of Rice (*Oryza sativa*)

**DOI:** 10.3390/ijms26062616

**Published:** 2025-03-14

**Authors:** Shuai Shao, Yuxin Wu, Lijie Zhang, Zhiyuan Zhao, Xianlong Li, Mingchong Yang, Haiyu Zhou, Songguo Wu, Lingqiang Wang

**Affiliations:** 1 State Key Laboratory of Conservation and Utilization of Subtropical Agricultural Biological Resources, Guangxi University, Nanning 530004, China; marshal@mail.hzau.edu.cn (S.S.); zhanglijie4421@163.com (L.Z.); 2317391029@st.gxu.edu.cn (X.L.); 2317301057@st.gxu.edu.cn (M.Y.); haiyuzhou@gxaas.net (H.Z.); 2 Guangxi Key Laboratory of Sugarcane Biology, College of Agriculture, Guangxi University, Nanning 530004, China; 3 College of Life Science and Technology, Huazhong University of Science and Technology, Wuhan 430074, China; u202213718@hust.edu.cn; 4 College of Informatics, Huazhong Agricultural University, Wuhan 430070, China; zhaozhiyuan@webmail.hzau.edu.cn; 5 Centre for Crop Science, Queensland Alliance for Agriculture and Food Innovation, The University of Queensland, St Lucia 4072, Australia

**Keywords:** AG peptides, stamen, pollen development, plant sterility, rice

## Abstract

Arabinogalactan proteins (AGPs) are complex proteoglycans present in plant cell walls across the kingdom. They play crucial roles in biological functions throughout the plant life cycle. In this study, we identified 43 gene members of the AG peptide (an AGP subfamily) within the rice genome, detailing their structure, protein-conserved domains, and motif compositions for the first time. We also examined the expression patterns of these genes across 18 tissues and organs, especially the different parts of the flower (anthers, pollen, pistil, sperm cells, and egg cells). Interestingly, the expression of some AG peptides is mainly present in the pollen grain. Transcription data and GUS staining confirmed that OsAGP6P—a member of the AG peptide gene family—is expressed in the stamen during pollen development stages 11–14, which are critical for maturation as microspores form after meiosis of pollen mother cells. It became noticeable from stage 11, when exine formation occurred—specifically at stage 12, when the intine began to develop. The overexpression of this gene in rice decreased the seed-setting rate (from 91.5% to 30.5%) and plant height (by 21.9%) but increased the tillering number (by 34.1%). These results indicate that AGP6P contributes to the development and fertility of pollen, making it a valuable gene target for future genetic manipulation of plant sterility through gene overexpression or editing.

## 1. Introduction

Arabinogalactan proteins (AGPs) belong to the cell wall glycoprotein family rich in hydroxyproline [1]. AGPs have common features, with glycan chains rich in arabinose, galactose, and other carbohydrates making up 90–98% (*w*/*w*) of the molecular structure, while a protein backbone high in hydroxyproline comprises 2–10% (*w*/*w*) [2,3,4]. AGPs are found in various tissues of many higher plants—including seeds, roots, stems, leaves, and inflorescences—where they play a crucial role in several physiological processes [5,6].

AGPs are classified into classical and non-classical types based on protein backbones [2]. Classical AGPs typically have three parts: an N-terminal signal peptide; a central domain rich in proline, alanine, serine, and threonine residues (PAST); and a C-terminal glycosylphosphatidylinositol (GPI) anchor [7]. However, some studies also categorize AGPs as classical AGPs with only the first two parts and lacking GPI [8]. Classical AGPs consist of two types: Lys-rich classical AGPs are separated by Lys-rich regions [9], and arabinogalactan (AG) peptides—with a length ranging from 50 to 90 amino acids—have an amino acid composition in which at least 35% of the residues are PAST [8,10]. There are also many chimeric AGPs featuring various conserved domains that can be classified into three main subfamilies: fasciclin-like AGPs (FLAs), phytocyanin-like AGPs (PAGs), and xylogen-like AGPs (XYLPs) [11,12,13,14,15]. Classical AGPs [16] and AG peptides [17,18] can be highly glycosylated (~90% *w*/*w*) [19], whereas chimeric AGPs are moderately glycosylated [20]. Furthermore, other AGPs cannot be classified into any of these three families [16,21].

AGPs feature extensive glycosylation, typically found on the Ser and Thr residues of the protein core through O-glycosylation. AGPs are attached to the cell membrane via the GPI anchor [16]. The signal peptide is essential for ensuring that AGPs are synthesized, processed, and delivered to the correct location (cell membrane or extracellular space) [2,22]. These distinct domains and the classification help us understand the diverse roles AGPs play in plant biology, including their involvement in cell signaling, development, and responses to environmental stresses (salt stress, drought, and pathogens) [21,23,24,25,26,27]. AGPs involve many cytological processes, including cell expansion, differentiation, and embryogenesis [28,29]. AGPs have gained considerable interest due to their intricate structure and function. However, their activity is also influenced by distribution, the timing of expression, and environmental conditions within cells or tissues [30,31]. Understanding their precise localization and expression timing could reveal how AGPs contribute to the structural integrity and developmental processes.

Several studies in *Arabidopsis* have revealed that AGPs are essential for pollen development and PT germination and growth [7,32,33,34]. Also, AGPs have been reported during pollen development in plants other than *Arabidopsis*, such as those in the *Brassica* genus or rice [7,34]. For example, the *OsFLA1* gene—from the FLA group—is found in spikelets, roots, stems, leaves, and leaf sheaths. It encodes a protein in the cytoplasm and nucleus that helps control pollen development [33]. In *OsAGP13*-RNAi plants, the cell walls in the flower spike axis are significantly thinner [35]. However, the information on the function of AG peptides in the rice pollen formation process is still limited. In addition, the mechanisms by which AGP influences pollen development are not fully understood. AGPs are part of the hormone response and signal transduction in plants. The expression of some *AGP* genes is affected by hormones like abscisic acid (ABA) and gibberellins (GAs) [36]. *AtAGP30* regulates ABA signaling pathways, and the *atagp30* mutant reduces the ABA-induced germination delay [37,38]. Calcium ion (Ca^2+^) signaling is crucial for cellular functions. Recent studies on AGP-Ca^2+^ oscillators have revealed the important role of AGP proteins in cells and their interaction with the auxin signaling network [39]. Despite these significant advances in understanding AGP functions in the dicot model plant *Arabidopsis thaliana*, their precise roles and their molecular mechanisms during pollen development in other plants remain elusive.

In a previous study, genomic studies in rice identified 69 AGPs, including 13 traditional classical AGPs, 15 AG peptides, 3 non-traditional AGPs, 3 early nodulin-like AGPs, 8 non-specific lipid transfer-like AGPs, and 27 fasciclin-like AGPs [10]. Some functions of these genes have been studied, while others remain unexplored. Given the advancements in genomic technologies (IIRGSP-1.0, MSU7, and RAP-DB) [40,41], particularly the recent release of the telomere-to-telomere (T2T) resolution of the Nipponbare rice genome [42], it is crucial to reassess and update our understanding of the AGP gene family in rice.

This study identified 43 classical AG peptide genes (designated *OsAGP1P* to *OsAGP43P*) from the *Oryza sativa japonica* genome. We systematically examined their gene structure, cis-acting elements, physicochemical properties, and conserved domains. The expression patterns of AG peptide genes across different growth stages and tissues were investigated. Specifically, OsAGP6P, one of the protein members, is found in the plasma membrane and is detectable during stages 11 to 14 of pollen development. Further transgenic expression confirmed its role in pollen development and various plant phenotypes. The findings will enhance the understanding of the structure and function of the AG peptides family in plants, which are vital for improving agricultural sustainability.

## 2. Results

### 2.1. Identification of AG Peptide Genes in Rice

**Figure 1:**
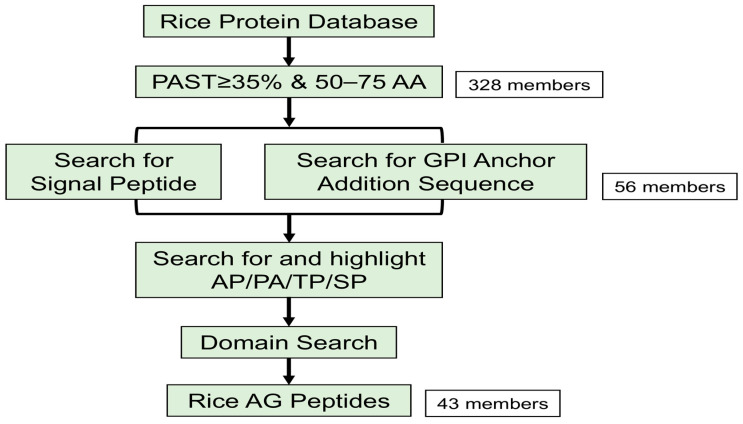
Workflow diagram for the identification of AG peptides in rice.

Considering the characteristics of AG peptides, we developed a detailed identification process (Figure 1). Initially, 328 potential AG peptides were identified in rice by searching for proteins between 50 and 75 amino acids long, with a biased amino acid composition of at least 35% PAST (Figure 1; Supplementary Materials, Table S2). Signal peptides are crucial for facilitating the glycosylation of AG peptides in the endoplasmic reticulum. Meanwhile, a glycosylphosphatidylinositol (GPI) anchor is required to anchor AG peptides to the plasma membrane. An additional screening process was performed on these 328 proteins to identify those that possess both a signal peptide and a GPI anchor (Supplementary Materials, Table S3). We also examined whether these proteins had dipeptide repeats (Ala–Hyp, Ser–Hyp, and Thr–Hyp), excluding proteins containing other domains (Supplementary Materials, Table S3). In total, 43 AG peptides were identified (Figure 1; Supplementary Materials, Table S2).

**Figure 2:**
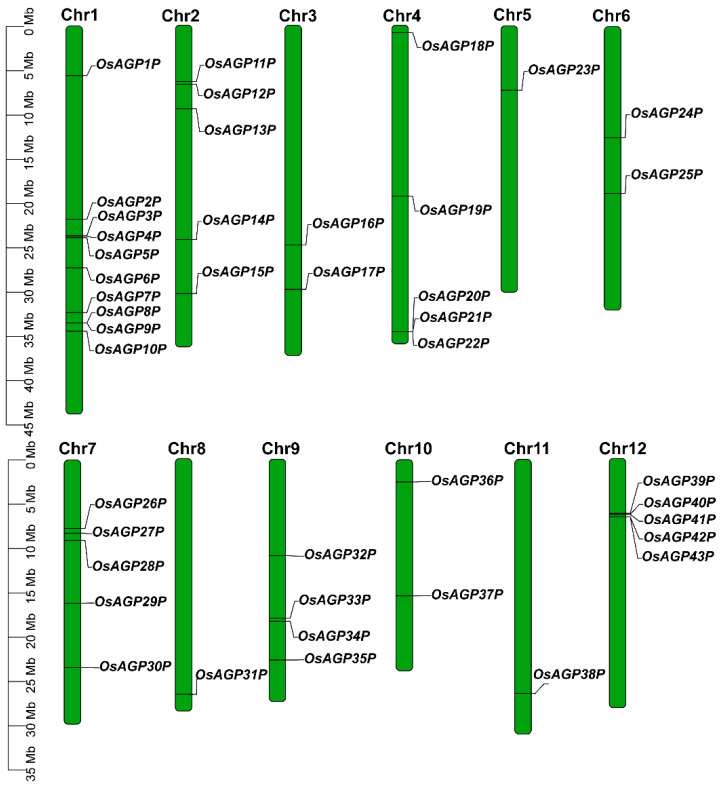
Chromosomal distribution of 43 AG peptide genes in rice. Chromosomes are represented by vertical bold bars with numerical orders at the top. The chromosome length scale (Mb) is indicated on the left side.

The 43 AG peptide genes identified were named *OsAGP1P* through *OsAGP43P* according to their chromosomal positions (Figure 2). These genes encode proteins with a length of 53–75 amino acids, with predicted molecular weights and isoelectric points from 5.09 kDa/4.00 to 7.79 kDa/12.10 (Table 1; Supplementary Materials, Table S4). The AG peptides were primarily predicted to localize in the cell membrane and nucleus. Some were also predicted to exist in cellular compartments like the Golgi apparatus, cytoplasm, and chloroplast (Supplementary Materials, Table S3). This multi-localization pattern suggests that these proteins may serve diverse roles in various physiological functions in plants.

### 2.2. Phylogenetic and Protein Structure Analyses of AG Peptide Proteins in Rice

**Figure 3:**
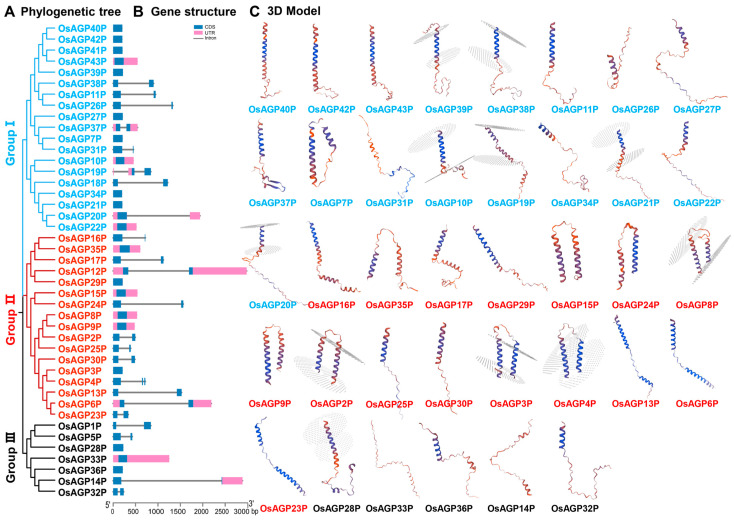
Phylogenetic relationship and 3D models of AG peptide proteins in rice: (**A**) phylogenetic tree; (**B**) gene structure of 43 AG peptide genes with a scale bar indicating 500 bp; (**C**) 3D structural models. Blue boxes indicate the coding regions (CDS), pink boxes indicate 5′ and 3′ and untranslated regions (UTR), black lines indicate introns.

An unrooted phylogenetic tree was generated by aligning AG peptide protein sequences to illustrate their phylogenetic relationships (Figure 3A). The AG peptide proteins were classified into three groups containing 19, 17, and 7 members, respectively (Figure 3A). The number of exons among the AG peptide genes ranges from one to three, with most having only one exon (Figure 3B). Our study presented a detailed three-dimensional (3D) structure of the AG peptide protein (Figure 3C). Group I proteins typically contain single α-helices connected to coils, transition through a β-turn, and subsequently link to additional coils. Unlike Group I proteins, Group II proteins have a more compact structure, typically consisting of two α-helices connected by a β-turn and linked to coils. Group III proteins contain two to three short α-helices, with β-turn junctions between them, and coils at the C-terminus or N-terminus. These findings clarify the evolutionary relationships and structural diversity of AG peptide proteins, enhancing our understanding of their classification and functions.

### 2.3. Identification of Cis-Regulatory Elements Within the Promoters of AG Peptide Genes

**Figure 4:**
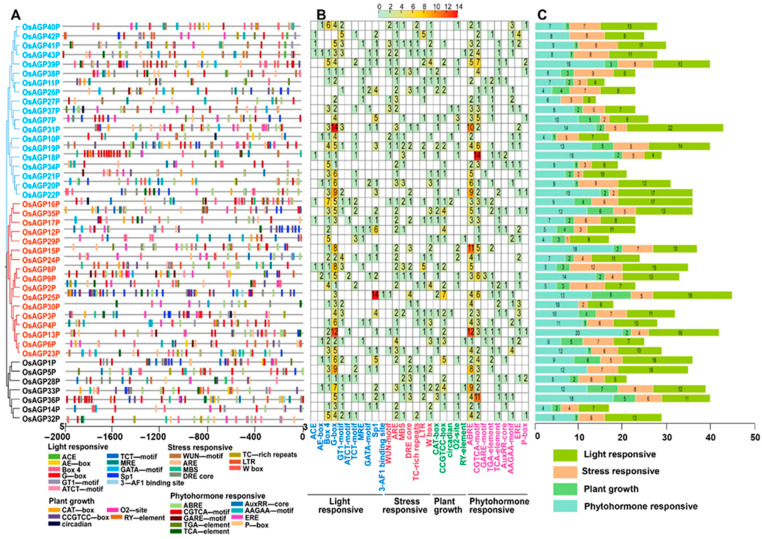
Cis-regulatory element analysis of the AG peptide genes: (**A**) analysis of cis-elements in the promoter region of AG peptide genes; (**B**) heatmap of the number of cis-elements, the different colors represent the number of cis-elements; (**C**) the sum of cis-elements for each category is shown as a histogram.

Cis-regulatory elements can significantly influence transcription regulation and the expression pattern of the associated genes. This study analyzed cis-regulatory elements based on the 2000-base-pair nucleotide sequences upstream of the start codon (ATG) for each AG peptide gene (Figure 4). In total, 32 types of elements were identified in the promoters of 43 AG peptide genes and categorized into four groups: light response (11), stress response (8), plant growth regulation (5), and phytohormone response (9) (Figure 4A,B). It was revealed that many identified elements were associated with light responsiveness, with the G-box element and Box 4 being the most frequently recognized (Figure 4B,C). The proportions indicate that the expression of the AG peptides gene was substantially affected by light.

Multiple plant hormone response elements, stress response elements, and plant growth regulation elements were also detected (Figure 4A). Elements associated with phytohormone responsiveness ranked second in frequency, while those concerning plant growth regulation were the least abundant (Figure 4C). These elements are essential for regulating gene expression during plant growth and development and responding to various stresses. The identified hormone response elements include the following: ABRE, TCA element, and AAGAA motif (abscisic acid); TGA element, AuxRP core, and p-box (auxin); CGTCA motif (MeJA); and ERE (ethylene). The stress response elements include the following: ARE elements (anaerobic induction); WUN motif (mechanical injury response); MBS and DRE core (drought stress response elements); and LTR (low-temperature response). Furthermore, various cis-elements related to plant growth—such as a CAT-box, CCGTCC-box, circadian, O2 site, and RY element—were also abundant. These findings suggest that AG peptide genes are regulated by hormones and environmental stresses, which govern plant development.

### 2.4. Expression Patterns of AG Peptide Genes in Multiple Tissues

**Figure 5:**
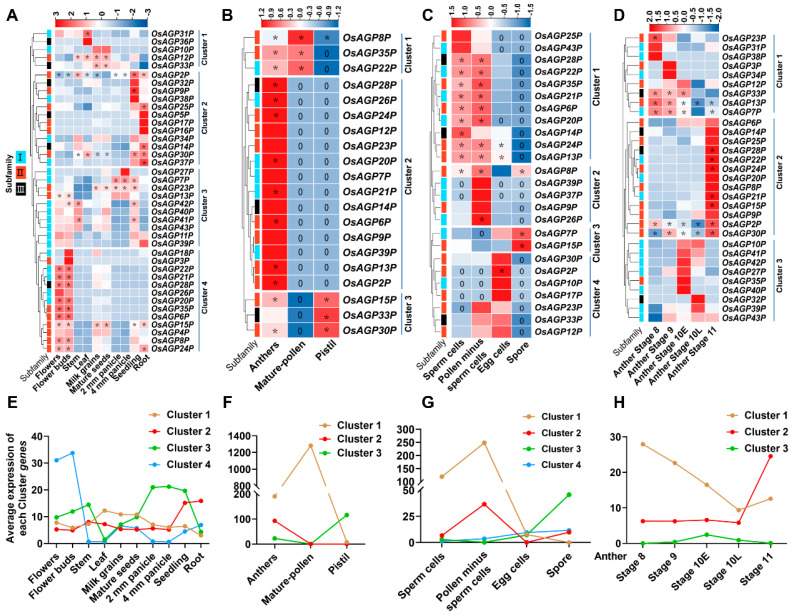
Hierarchical clustering of the expression profiles of the AP peptide genes in diverse tissues at different stages. (**A**,**E**) Expression of 43 AG peptide genes in various tissues exhibited divergent patterns. (**B**,**F**) Expression of 43 AG peptide genes in flower parts: anther, mature pollen, and pistil. (**C**,**G**) Expression of 43 AG peptide genes in sperm cells, pollen minus sperm cells, egg cells, and spores. (**D**,**H**) Expression of AG peptide genes at stages 8 to 11 of the anther development. The expression values of each point in (**E**–**H**) were calculated from the mean values of the expression of all members in each cluster. The heatmap was created based on the log_2_ (TPM) values of AP peptide genes and normalized by row. A TPM value above 20 displays abundant genes and is marked with “*”. Differences in gene expression are colored, with red for high expression and blue for low expression.

The expression profiles of 43 AG peptide genes in various tissues exhibited divergent patterns and were classified into some clusters accordingly (Figure 5A,E). Most genes showed tissue-specific expression functions. Genes in Clusters 1, 2, and 3 were constitutively expressed. The genes in Cluster 1 (*OsAGP12P*/*OsAGP31P*/*OsAGP33P*) displayed higher expression levels in the leaves, milk grains, and mature seeds. The Cluster 2 genes (*OsAGP2P*/*OsAGP9P*/*OsAGP14P*/*OsAGP25P*/*OsAGP30P*/*OsAGP37P*) exhibited higher expression levels in the seedlings and roots. Cluster 3 genes (*OsAGP7P*/*OsAGP13P*/*OsAGP23P*/*OsAGP41P*/*OsAG42P*) showed higher expression in the stems, panicles, and seedlings. By contrast, Cluster 4 genes (*OsAGP6P*/*OsAGP8P*/*OsAGP15P*/*OsAGP20P*/*OsAGP21P*/*OsAGP22P*/*OsAGP24P*/*OsAGP28P*/*OsAGP35P*) presented preferential expression in the flowers and flower buds, with expression levels significantly higher than other tissues.

We further analyzed the expression of these AG peptide genes in different parts of a flower, including the anther, mature pollen, and pistil (Figure 5B,F). Interestingly, we found that all genes could be detected in the anther. Cluster 2 genes (*OsAGP2P*/*OsAGP6P*/*OsAGP13P*/*OsAGP20P*/*OsAGP21P*/*OsAGP24P*/*OsAGP26P*/*OsAGP28P*) were specifically expressed in the anther. In addition to the anther, the Cluster 3 genes (*OsAG15P*/*OsAGP30P*/*OsAGP33P*) and Cluster 1 genes (*OsAG8P*/*OsAGP22P*/*OsAGP35P*) were expressed in the pistil and mature pollen, respectively. Notably, the expression levels of these genes were high overall.

Furthermore, we investigated the expression of these AG peptide genes in sperm cells, pollen minus sperm cells, egg cells, and spores (Figure 5C,G). Cluster 1 genes (*OsAGP6P*/*OsAGP13P*/*OsAGP14P*/*OsAGP20P*/*OsAGP21P*/*OsAGP22P*/*OsAGP24P*/*OsAGP28P*/*OsAGP35P*) exhibited higher expression levels in sperm cells and pollen minus sperm cells. Cluster 2 genes (*OsAGP8P*/*OsAGP26P*) demonstrated preferential expression in pollen minus sperm cells. Cluster 3 genes (*OsAGP7P*/*OsAGP115P*) showed preferential expression in spores. In contrast, Cluster 4 genes showed preferential expression in egg cells.

Finally, we investigated the expression of these genes in anthers from stage 8 to stage 11 (Figure 5D,H). Cluster 1 displays a relatively high expression in one or two points between stage 8 and the early phase of stage 10, followed by a decrease at the later phase of stage 10 until stage 11. In contrast, Cluster 2 demonstrated lower expression during stage 8 to the later phase of stage 10 but was significantly higher at stage 11. Cluster 3 was expressed much higher in the early or later phases of stage 10. Obviously, the three clusters showed a distinctive complementary expression in anthers.

### 2.5. Subcellular Localization of the OsAGP6P Protein and the GUS Staining of OsAGP6P in Tissues and Organs

In this study, we found that one of the gene members, *OsAGP6P*, is preferentially higher in flowers, specifically in sperm cells and pollen (Figure 5). This result indicates that *OsAGP6P* may play a role in anther development and pollen formation in rice. Therefore, the *OsAGP6P* gene was chosen for further investigation.

**Figure 6:**
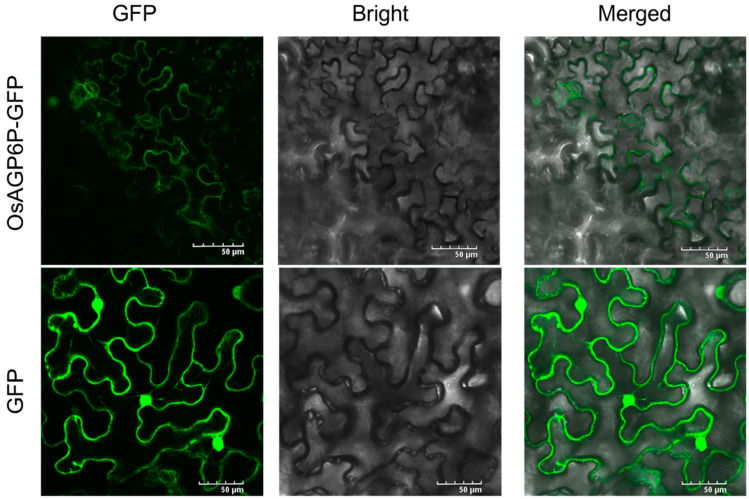
Subcellular location of OsAGP6P protein in *Nicotona benthamiana*. GFP is the empty vector pD1301S; OsAGP6P-GFP is the recombinant vector *OsAGP6P-*pD1301S. Scale bars, 50 μm.

The localization of the OsAGP6P protein was predicted in the plasma membrane using WoLF PSORT and Cell-Ploc 2.0. This result was verified as we found that GFP fluorescence was observed at the plasma membrane of tobacco leaf epidermal cells after the OsAGP6P-GFP fusion protein was transiently expressed in the leaves of four-week-old tobacco (*Nicotiana benthamiana*) (Figure 6). The plasma membrane localization of the OsAGP6P protein indicates its potential role as a small secreted protein.

**Figure 7:**
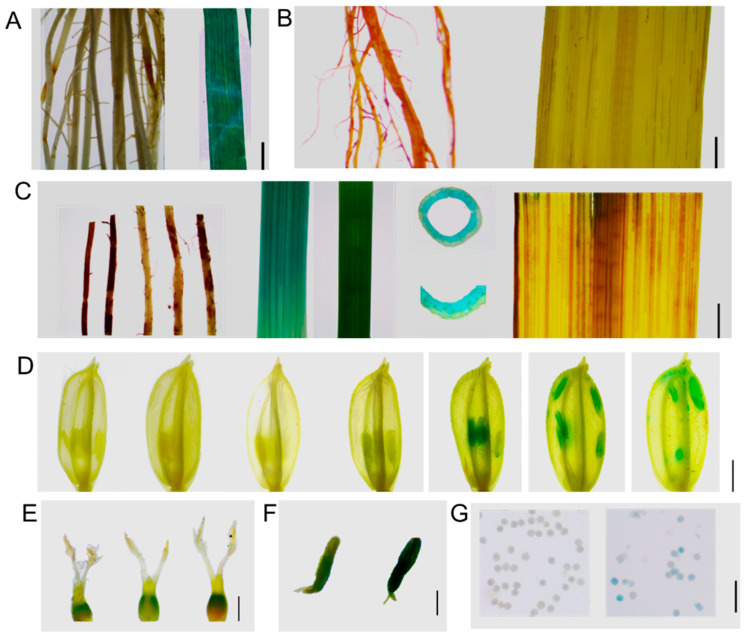
Histochemical GUS staining of *ProOsAGP6P-GUS* plants: (**A**) Stained tissues of roots and leaves at the seedling stage. Scale bar, 5 mm. (**B**) Stained tissues of roots and leaves at the vegetative stage. Scale bar, 5 mm. (**C**) Stained tissues of roots, basal internodes, third internodes, third internode slices, and leaves at heading stage. Scale bar, 5 mm. (**D**) Stained floral tissues from stages 8 to 14 of anther development. Scale bar, 2 mm. (**E**) The stained tissues of pistils, with stamens removed, show the 9th, 11th, and 13th stages of anther development from left to right. Scale bar, 500 μm. (**F**) Stained tissues of the anther, from left to right, represent the 11th and 13th stages of anther development. Scale bar, 200 μm. (**G**) Stained pollen tissues, shown from left to right, represent the 11th and 13th stages of anther development. Scale bar, 100 μm.

Additionally, to investigate the expression of *OsAGP6P* at the tissue level further, GUS staining was performed on the tissues and organs at different stages of the *ProOsAGP6P*::GUS transgenic plants (Figure 7). Except for minimal staining in the young leaves of 14-day-old seedlings, no staining was observed in the roots or leaves at other vegetative stages (Figure 7A,B). Staining was not observed in the roots and leaves during the reproductive stage; however, it was evident in the basal internodes and the third internode of the rice stem. Transverse sections of the third internode revealed stronger staining in the vascular bundles of the stem (Figure 7C). Though insignificant, GUS staining in rice flowers began at stage 11 of anther development. The staining reached its darkest color at stage 12 (when microspores are formed after the meiosis of pollen mother cells) and continued until stage 14, when the rice flower fully bloomed (Figure 7D). These results indicate that *OsAGP6P* is expressed later during anther development. In detail, GUS staining can be observed in the stamens, specifically in the tapetum layer but not in the pollen at stage 11. However, at stage 13, slight GUS staining can be observed in pollen (Figure 7F,G). In contrast, the pistil showed no staining during anther development (Figure 7E). The findings indicate that the OsAGP6P protein primarily functions in the anther cell wall layer, although its effects on pollen tube growth cannot be excluded.

### 2.6. Phenotypic Observation of OsAGP6P Overexpression Plants

**Figure 8:**
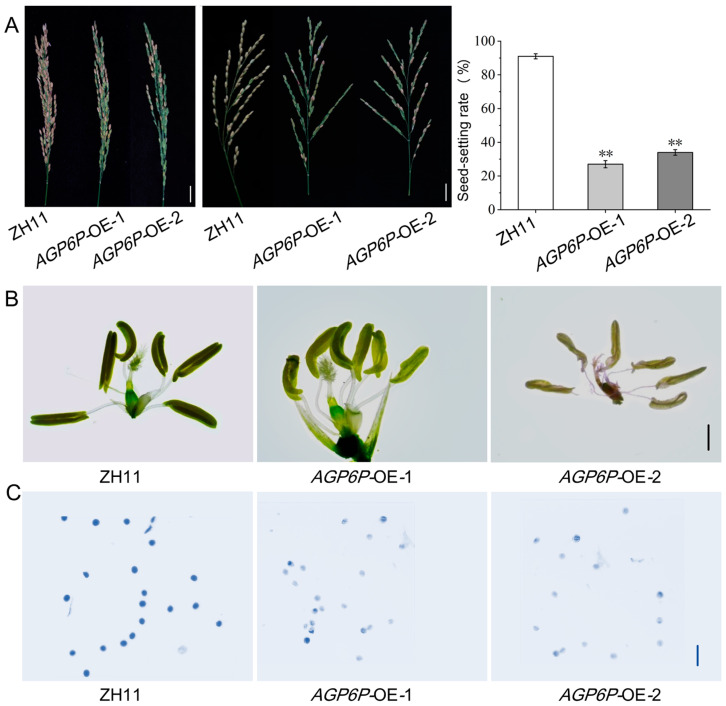
Spikelets, seed-setting rate, florets, and pollen viability of rice with overexpression of *OsAGP6P*: (**A**) From left to right, spikelets of T_1_ generation rice overexpressing *OsAGP6P*, spikelets of T_2_ generation rice overexpressing *OsAGP6P*, seed-setting rate of T_2_ generation rice overexpressing *OsAGP6P*. Values represent the means, error bars depict SE (Scale bar, 10 cm. Student’s *t*-test, n = 10, ** *p* < 0.01). (**B**) Flowers of T_2_ generation rice overexpressing *OsAGP6P*. Scale bar, 1 mm. (**C**) I_2_-KI staining of pollen from T_2_ generation rice overexpressing *OsAGP6P*. Scale bar, 100 μm.

Plants from two positive independent overexpression lines (*AGP6P*-OE-1 and *AGP6P*-OE-2) and wild-type (WT) ZH11 were planted and grown in the field to examine the panicles at the mature stage. All positive plants exhibited a reduced seed-setting rate, while the negative plants exhibited no significant difference compared to the wild type (Figure 8A). These results indicated that overexpression of the *OsAGP6P* impaired the seed-setting rate and caused plant sterility in rice.

To further investigate the impact of *OsAGP6P* overexpression on the fertility of the plants, seed-setting rates were calculated, and the flowers of the two OE lines were examined. It was found that the overexpression of *OsAGP6P* hampered reproductive development in transgenic plants as they exhibited many green and empty grains, while the WT plants had golden-yellow seeds. The seed-setting rates were significantly lower in the two OE lines compared to the WT (from 91.5% to 27.2% and 34.1%) (Figure 8A). The anthers of the two OE lines seemed paler in color, had fewer filled pollen sacs, and were slightly curled (Figure 8B). After I2-KI staining, nearly all the pollen from the OE lines did not appear blue, indicating that they were inactive (Figure 8C).

**Figure 9:**
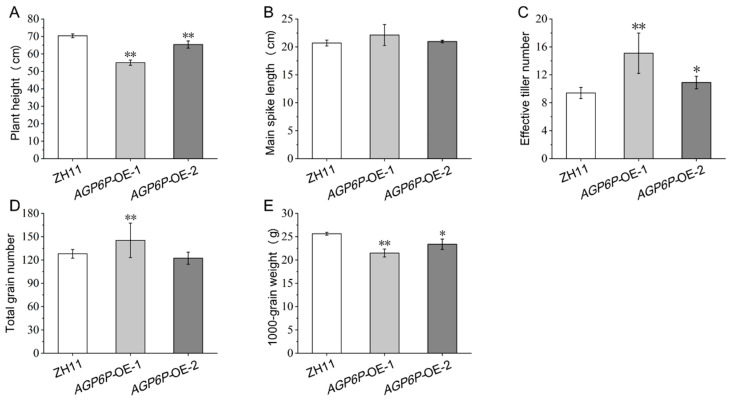
The agronomic traits of ZH11 and OE lines (*AGP6P*-OE-1, *AGP6P*-OE-2) plants. (**A**) Plant height of ZH11 and OE lines. (**B**) Main spike length of ZH11 and OE lines. (**C**) Effective tiller number of ZH11 and OE lines. (**D**) Total grain number of ZH11 and OE lines. (**E**) 1000-grain weight of ZH11 and OE lines. Three repetitions for 10 plants each (3 replicates × 10 individuals). Values represent the means, error bars depict SE (Student’s *t*-test, n = 10, * *p* < 0.05, ** *p* < 0.01).

To explore the effect of *OsAGP6P* on rice growth and development, several agronomic traits—including plant height, main spike length, effective tiller number, total grain number, and 1000-grain weight—were analyzed. No significant difference in main spike length was found between the OE lines and the WT ZH11 (Figure 9B). However, the OE lines showed a noticeable reduction in plant height and a significant increase in the effective tiller number compared to the WT ZH11 (Figure 9A,C). Compared with the wild type, the plant height of *AGP6P*-OE-1 decreased by 21.9%, while the effective tiller number increased by 60.6%. The *AGP6P*-OE-1 line had the shortest plant height (55.0 cm) and the highest effective tiller number (15.1), while the WT ZH11 had only 9.4 effective tillers on average (Figure 9C). In addition, the 1000-grain weights were significantly lower in the two OE lines, with the values 21.5g and 23.4g, respectively (Figure 9E). The results indicate that many yield components were affected by the overexpression of the gene *OsAGP6P* in rice.

## 3. Discussion

AGPs are subdivided according to the structure of their protein backbone into classical, lysine-rich, AG peptide, fasciclin-like (FLAs), non-classical, and chimeric AGPs [14,43,44,45]. The AG peptides are classical and possess complete structural features yet they are relatively under-researched. A genome-wide analysis of the AGP gene family should be performed as a first step in researching AGP gene families. Previous studies identified AGPs in rice but the information was fragmented and the number of members in each subfamily remained to be corrected.

In this study, with the recent availability of the latest genomic data from rice and the use of the “Python” programming language, AP peptides were identified. We tested various parameters to achieve the desired results. As expected, the number of AG peptides in this study was greatly increased compared to previous studies [10]. This computational methodology developed in this study can effectively differentiate PAST-rich proteins from others by applying specific thresholds (e.g., >35%) and can also be adapted for use with other species.

Based on these precise protein sequences, the diversity of AG peptides in rice can be analyzed in depth, and the phylogenetic analysis categorized them into three distinct groups. Additionally, we systematically elucidated the compositional characteristics and 3D structures of the AG peptides. AG peptides primarily consist of 1–2 α-helices, essential for protein recognition and binding, and all have a signal peptide that targets protein precursors to the endoplasmic reticulum (ER). In addition, we found that some AG peptides contain a GPI lipid anchor that was reported to tether the AGP molecules to the outer leaflet of the plasma membrane [4]. Many transmembrane proteins at the cell membrane feature α-helices that embed in the lipid bilayer, forming transmembrane channels or transporters. These structures are actively involved in the transmembrane transport of substances and the transmission of signals [46]. Some previous studies reported AGP proteins localized to multiple cellular structures, including the cell wall and the protoplast, or attached to the cell membrane via a GPI anchor at their C-terminus [47,48]. Lamport and Várnai [49] suggested that AGPs act as Ca^2+^ capacitors, indicating their role in Ca^2+^-dependent signaling pathways at the plasma membrane level. Another signaling pathway where AGPs may be significant is associated with the cleavage of their GPI anchor by phospholipase C [6,50]. Therefore, emphasizing structure and classification in this study was crucial for understanding AG peptide function in rice. It is important to note that the molecular size of AG peptides identified in this study ranges from approximately 5.09 kDa to 7.79 kDa. Thus, the AG peptides identified in our study can also be classified as small-molecule-secreted proteins (SSPs). SSPs are less than 250 amino acids (aa) in length and can be actively transported out of plant cells [51,52]. They regulate plant development and stress responses; studying them boosts plant resilience and productivity, making them a key research focus [53]. Therefore, our study serves as a valuable resource for investigating AG peptides in rice, revealing their role in plant development and male sterility while highlighting their potential targets for genetic engineering as a type of SSP.

Furthermore, the expression pattern of AG peptide genes was dynamically revealed during flowering, especially in the floral tissues as AGPs were frequently associated with pollen development [54,55]. Our study examined AG peptides in rice, particularly their roles in male reproductive development, establishing a crucial foundation for future research on the functions of AG peptides in rice development and stress response. We specifically demonstrated that *OsAGP6P*, a member of the rice AG peptide gene family, is associated with pollen development. Transcription data and GUS staining confirmed that the OsAGP6P protein is present in the stamen during stages 11–14 of pollen development. This generally represents critical transitions in pollen maturation, when microspores are formed after the meiosis of pollen mother cells. It was detectable from stage 11 (the late microspore stage when the exine formation occurs)—specifically at stage 12 (when the inner pollen wall, or intine, begins to develop beneath the exine). The results were consistent with the functions of AGPs, which have been reported to play a crucial role in anchoring primexine, followed by the formation of nexine and intine, as well as cellulose deposition, thereby providing essential structural support to the pollen grain [34]. It was shown that a change in the AGP expression pattern becomes evident from the early stages of the male gametophyte development [34]. In *Arabidopsis*, AGPs first appear in the cell walls of PMCs and endothelial cells. After meiosis, AGPs are found in the cytoplasm and primary cell wall of tetrads, forming a reticulated layer above the protectum of tetrads as well as in the locule-facing cell wall of the tapetum [56]. Although our study indicates that *OsAGP6P* is expressed at stages 11 and 14, we cannot dismiss the possibility that *OsAGP6P* is also expressed earlier (in PMCs) and contributes to pollen tube growth. Some studies have shown that variations in GPI biosynthesis affect pollen germination and pollen tube extension. For instance, the MAC207 antibody detects AGP proteins on the surface of mature pollen grains near the inner side of the plasma membrane [57]. AGP proteins were also observed in the cell walls of pollen tubes in *Arabidopsis* cultured in vitro [58]. AGP proteins profoundly impact the development of floral organs and ensure the proper timing of pollen grain germination and correct directional growth of pollen tubes [58,59]. Further work requires sensitive detection techniques like RNA in situ hybridization or immunolocalization using monoclonal antibodies specific to AGP sugar epitopes.

Our study has limitations that need future addressing, for example: (I) It is necessary to determine whether the OsAGP6P protein impacts pollen recognition at the stigma and the germination of pollen tubes, and whether it has functions in vegetative tissues. GUS staining also revealed that OsAGP6P expression occurs in leaves during the seedling stage and at the stem base during heading, potentially influencing agricultural traits in transgenic plants. (II) The precise mechanism underlying its mode of action still needs to be clarified. It has been reported that some transcription factors, such as novel microgametophyte defective mutant 1 (NMDM1; AT5G09250), can control the expression of AGPs for proper intine formation [60]. Are there any interacting partners that could explain their role in ensuring the proper timing of pollen grain development? Continued research in these areas is crucial for a deeper understanding of AGPs’ roles and could have implications for plant reproductive biology and crop improvement strategies.

## 4. Materials and Methods

### 4.1. The Identification of AG Peptide Proteins in Rice

The search criteria for AGPs referred to the guidelines [8,10,43,45,61]. A Python script was written to screen proteins with amino acid lengths between 50 and 75 and to calculate the PAST (Pro, Ala, Ser, Thr) percentage (greater than or equal to 35%, amino acid bias) for the candidate proteins (Supplementary Materials S1). The sequences of all annotated proteins in Nipponbare (*Oryza sativa japonica*) were downloaded from the Rice Super Pan-genome Information Resource Database (RiceSuperPIRdb, http://www.ricesuperpir.com/ (accessed on 7 November 2024)) [42]. Python 3.13.1 for Windows, macOS, UNIX, and Linux operating systems (https://www.python.org/ (accessed on 7 November 2024)) was used in this study.

The presence of N-terminal signal peptides of AG peptide proteins was predicted by the SignalP 5.0 Server (https://services.healthtech.dtu.dk/services/SignalP-5.0/ (accessed on 10 November 2024)) [62]. The presence of a C-terminal GPI anchor of AG peptide proteins was predicted using the GPI-SOM (http://gpi.unibe.ch, accessed on (accessed on 8 November 2024)) and the BIG-PI Plant Predictor (http://mendel.imp.ac.at/gpi/plant_server.html (accessed on 12 November 2024)).

After removing incomplete domains, the remaining proteins were chosen as AG peptide proteins for further analysis. The biochemical characteristics of AG peptide proteins, including molecular weight, instability index, isoelectric point (pI), and amino acid counts, were analyzed using TBtools [63]. The protein sequences for each gene were submitted to the website (http://www.csbio.sjtu.edu.cn/bioinf/plant-multi (accessed on 10 November 2024)) for predicting subcellular locations, following a previous study [64].

### 4.2. Phylogenetic Analysis, the Motifs Prediction, and the Three-Dimensional (3D) Structure Models of AG Peptide Proteins

Multiple alignments of AG peptide protein sequences in *Oryza sativa* were generated using MEGA 11 software [65]. Phylogenetic trees were constructed using a neighbor-joining algorithm and drawn using MEGA 11 software. Bootstrapping was implemented with 1000 replicates. Based on their sequence homology, all AG peptide proteins were clustered into distinct clades according to different subfamilies. The motifs within the AG peptide proteins were investigated using the Multiple Em for Motif Elicitation (MEME) (https://meme-suite.org/meme/ (accessed on 10 November 2024)). Three-dimensional (3D) protein models were constructed using the SWISS-MODEL platform (https://swissmodel.expasy.org (accessed on 10 November 2024)) [66,67].

### 4.3. Chromosomal Mapping, Gene Structure Analysis, and Promoter’s Cis-Element Analysis of AG Peptide Genes

TBtools software (version 2.019) was used to obtain and visualize the chromosomal location information of rice AG peptide genes. The intron–exon structure of the AG peptide genes was determined using TBtools, facilitating the integrated visualization of the motifs and the gene structure map [63]. The rice genome annotation file was downloaded from the Rice Super Pan-genome Information Resource Database (RiceSuperPIRdb, http://www.ricesuperpir.com/ (accessed on 7 November 2024)) [42]. The program Gtf/Gff3 Sequences Extract in TBtools software was used to extract upstream 2000-base-pair promoter sequences of the 43 AG peptides genes. AG peptides utilize “ep” or a similar variant as the suffix for the term “AGP”. This study employs the suffix “P” to differentiate the AG peptides from the traditional AGP members. Consequently, the genes of the AG peptide subfamily are referred to as *AGP1P* through *AGP43P* in this study. The upstream 2000-base-pair promoter sequences of each member were submitted to PlantCARE (http://bioinformatics.psb.ugent.be/webtools/plantcare/html/ (accessed on 7 November 2024)) [68] to forecast cis-elements and their potential function. The TBtools software was used to visualize the cis-element composition [63].

### 4.4. Expression Pattern Analysis of AG Peptide Genes

The transcriptomic data of 43 AG peptide genes were available on the Plant Public RNA-seq Database (PPRD) (https://plantrnadb.com/ (accessed on 10 November 2024)) [69]. Co-expression analysis was performed utilizing Euclidean distances in conjunction with the hierarchical clustering approach known as complete linkage clustering, with the analyses executed using TBtools [63]. To depict the overall expression profiles of each group, the average expression values of all co-expressed members within a cluster were calculated and represented in a line chart format.

### 4.5. Subcellular Localization Analysis of OsAGP6P Protein

The coding sequence (CDS) of the *OsAGP6P* gene (excluding the stop codon) was amplified from the rice variety ZH11. Under the control of the CaMV35S promoter, it was fused with the enhanced green fluorescent protein (eGFP) in the vector pD1301S to construct the plasmid *OsAGP6P-eGFP*. The plasmid was injected into *Agrobacterium tumefaciens* (EHA105) and transformed into the epidermal cells of 4–6-week-old tobacco (*Nicotiana benthamiana* L.) leaves according to the method of Kokkirala et al. [70]. After 36–48 h of injection, the subcellular localization of protein OsAGP6P-eGFP was observed using a FluoView FV3000 confocal microscope (Olympus, Tokyo, Japan).

### 4.6. The Construction and the Transformation of the Recombinant Vector pCAMBIA1381Z-OsAGP6P and GUS Staining of the Tissues from Transgenic Plants

The promoter region of the *OsAGP6P* gene (2000 bp upstream of the ATG) was obtained from the Rice Genome Annotation Project (RGAP) database. It was amplified from the DNA of ZH11 using the *OsAGP6P*-GUS primers (Supplementary Materials, Table S1). *BamH* I and *EcoR* I restriction sites were selected for cloning, and the amplified product was fused with the GUS-containing vector pCAMBIA1381Z to construct the recombinant expression vector pCAMBIA1381Z-*OsAGP6P*. The recombinant vector was inserted into DH5α competent cells for plating, and the colony was singled out for sequencing. After sequencing, the vector was transformed into the ZH11 callus via Agrobacterium-mediated transformation. Positive transgenic lines were identified by PCR using the hygromycin resistance gene detection primers (HygF and HygR) and further confirmed by *β-glucuronidase* (*GUS*) reporter gene-specific primers (*OsAGP6P*-GUS-IdentifyF and *OsAGP6P*-GUS-IdentifyR) (Supplementary Materials, Table S1). The primers were designed with Primer 5.0 and synthesized by Beijing Tsingke Biotechnology Co., Ltd.

The qualitative detection of the GUS reporter gene for *OsAGP6P* was conducted following the method of Lee and Schöffl [71]. The expression of *OsAGP6P* in different tissues was determined in the *ProOsAGP6P*::GUS (the β-glucuronidase (GUS) reporter gene driven by the promoter of *OsAGP6P* gene) transgenic lines. The expression of the *β-glucuronidase* (*GUS*) reporter gene was observed by histochemical staining [72]. For GUS staining, tissue samples were immersed in 90% acetone for 30 min, then stained in 5-bromo-4-chloro-3-indolyl-glucronide solution at 37 °C overnight. Then, the samples were transferred to 75% ethanol to remove chlorophyll and were observed under a stereomicroscope [73].

### 4.7. The Construction and the Investigation of Pollen Sterility of OsAGP6P Overexpression Transgenic Plants

For generating *OsAGP6P* overexpression lines, the coding sequence (CDS) of the *OsAGP6P* gene was amplified using the cDNA of ZH11 anther as the template, leaving out the stop codon and 20 base pairs from both the upstream and downstream parts of the cloning site. Primers were designed using the CEII tool, and *Bam*HI and *Sma*I restriction enzyme sites were used (Supplementary Materials, Table S1). Gel electrophoresis checked the fragment size. Then, the fragment was cloned into the intermediate vector using a pEASY-Blunt Cloning Kit. Finally, the fragment was linked into the linearized binary expression vector pRHVcGFP, which has the enhanced green fluorescent protein (eGFP) sequence. The recombinant plasmid pRHVcGFP-OsAGP6P was introduced into the Zhonghua 11 (ZH11) by Agrobacterium-mediated transformation [74]. The positive transgenic lines were identified by PCR using the gene-specific primer combination *OsAGP6P*-pRYJ (Supplementary Materials, Table S1).

### 4.8. The Agronomic Traits and Statistical Analysis of OsAGP6P Overexpression Transgenic Plants

All overexpression plants and WT were grown at the experimental farm of Guangxi University (108° E, 22° N) in the 2023 rice growing season in Nanning, China. Seedlings approximately 35 days old were transplanted to a single-row plot, with a distance of 17 cm between plants within a row, and the rows were 27 cm apart. The field management followed essentially the normal agricultural practice. Pollen collected from *OsAGP6P*-overexpressing plants was stained with an I_2_-KI solution and examined using a stereomicroscope (Leica S8 APO, Wetzlar, Germany) to observe pollen viability. If the color of pollen grains appears blue–black, it indicates a high starch content and strong pollen vitality. The proportion of pollen in a microscopic field that can be stained can be calculated (%) using at least three microscopic fields from each observed sample. Only the 10 plants in the middle of each row were selected to measure agronomic traits, including plant height, main spike length, effective tiller number, total grain number, seed-setting rates, and 1000-grain weight. The method for determining the 1000-grain weight involved counting 100 fully developed and well-colored rice seeds, weighing them, and then converting the weight to the 1000-grain weight. Photos of the mature spikes and florets were taken during the measurements.

### 4.9. Statistical Analysis

Each experiment was conducted thrice, with data presented as means ± standard deviation (SD) from independent assays. Student’s *t*-test in GraphPad Prism version 8.0 was used to assess significance at the *p* < 0.01 level. Bar charts were generated using Origin 2018.

## 5. Conclusions

Arabinogalactan proteins (AGPs) are complex proteoglycans in nearly all plant organs, crucial for various biological functions in the plant life cycle. Here, we provide a computational methodology for screening AG peptides in rice and describe the classification, characteristics, and expression patterns of 43 AG peptides in detail. A focus is mainly placed on *OsAGP6P*, preferentially expressed in pollen during the later stages of anther development, encoding a small protein at the plasma membrane. Overexpressing *OsAGP6P* in rice reduced the seed-setting rate, pollen vitality, plant height, and 1000-grain weight while increasing the number of effective tillers, indicating its significant role in pollen ontogenesis and fertility. This understanding is essential for utilizing AG peptides in developing male-sterile lines, which are crucial for producing hybrid seeds.

## Figures and Tables

**Table 1:** Identification and characterization of the AG peptides genes in rice.

AGIS ID	Locus ID	Gene Name	PAST (%)	SP	GPI	AP/PA/SP/TP Repeats	AA Size
AGIS_Os01g009100	LOC_Os01g10550	*OsAGP1P*	35.62	Yes	No	0/2/0/0	73
AGIS_Os01g032060	LOC_Os01g37950	*OsAGP2P* (*OsAGP14*)	51.43	Yes	No	4/2/0/0	70
AGIS_Os01g034750	LOC_Os01g40880	*OsAGP3P*	40.00	Yes	Yes	0/1/1/0	70
AGIS_Os01g034820	LOC_Os01g40950	*OsAGP4P* (*OsAGP15*)	40.28	Yes	No	3/3/0/0	72
AGIS_Os01g035100	LOC_Os01g41230	*OsAGP5P*	44.93	Yes	No	2/1/1/0	69
AGIS_Os01g039860	LOC_Os01g46850	*OsAGP6P* (*OsAGP6P*)	40.58	Yes	Yes	3/2/0/0	69
AGIS_Os01g047750	LOC_Os01g55220	*OsAGP7P* (*OsAGP17*)	47.14	Yes	Yes	4/3/0/0	70
AGIS_Os01g049500	LOC_Os01g57030	*OsAGP8P* (*OsAGP18*)	58.82	Yes	Yes	3/2/0/0	68
AGIS_Os01g049510	LOC_Os01g57040	*OsAGP9P* (*OsAGP19*)	57.35	Yes	Yes	4/3/0/0	68
AGIS_Os01g051100	LOC_Os01g58590	*OsAGP10P*	37.50	Yes	No	0/2/0/0	64
AGIS_Os02g010250	LOC_Os02g12150	*OsAGP11P*	42.25	Yes	No	0/2/3/0	71
AGIS_Os02g010720	LOC_Os02g12654	*OsAGP12P*	40.91	Yes	No	2/0/1/0	66
AGIS_Os02g014470	LOC_Os02g16500	*OsAGP13P* (*OsAGP20*)	38.36	Yes	Yes	4/0/1/0	73
AGIS_Os02g035140	LOC_Os02g39290	*OsAGP14P*	43.75	Yes	No	1/1/2/0	64
AGIS_Os02g044090	LOC_Os02g48710	*OsAGP15P* (*OsAGP21*)	49.28	Yes	Yes	2/2/0/0	69
AGIS_Os03g036830	LOC_Os03g42990	*OsAGP6P*	43.84	Yes	Yes	0/1/1/0	73
AGIS_Os03g043930	LOC_Os03g50720	*OsAGP17P*	38.67	Yes	No	1/0/0/1	75
AGIS_Os04g001290	LOC_Os04g02380	*OsAGP18P*	39.13	Yes	No	2/1/0/1	69
AGIS_Os04g027720	None	*OsAGP19P*	38.81	Yes	No	1/1/1/0	67
AGIS_Os04g050800	LOC_Os04g57260	*OsAGP20P*	62.32	Yes	No	4/4/2/0	69
AGIS_Os04g050820	LOC_Os04g57270	*OsAGPp21P*	60.29	Yes	No	4/4/2/0	68
AGIS_Os04g050830	LOC_Os04g57280	*OsAGP22P*	57.97	Yes	No	4/4/2/0	69
AGIS_Os05g010800	LOC_Os05g12580	*OsAGP23P*	43.28	Yes	Yes	2/1/1/0	67
AGIS_Os06g019180	LOC_Os06g21410	*OsAGP24P* (*OsAGP25*)	45.95	Yes	No	3/3/0/0	74
AGIS_Os06g027160	LOC_Os06g30920	*OsAGP25P*	50.94	Yes	No	3/4/0/0	53
AGIS_Os07g011720	LOC_Os07g13319	*OsAGP26P*	41.79	Yes	No	0/2/0/0	67
AGIS_Os07g012700	LOC_Os07g14360	*OsAGP27P*	43.66	Yes	No	1/2/2/0	71
AGIS_Os07g013810	LOC_Os07g15530	*OsAGP28P*	36.00	Yes	No	0/1/1/0	75
AGIS_Os07g023740	LOC_Os07g27410	*OsAGP29P*	45.71	Yes	No	3/0/3/0	70
AGIS_Os07g034440	LOC_Os07g38630	*OsAGP30P* (*OsAGP27*)	40.63	Yes	No	4/3/0/0	64
AGIS_Os08g038920	LOC_Os08g41860	*OsAGP31P*	41.67	Yes	No	0/1/0/1	72
AGIS_Os09g019700	LOC_Os09g11780	*OsAGP32P*	41.67	Yes	No	1/1/2/1	60
AGIS_Os09g029540	LOC_Os09g22510	*OsAGP33P*	48.44	Yes	No	1/2/0/0	64
AGIS_Os09g029970	LOC_Os09g23430	*OsAGP34P*	37.88	Yes	No	2/0/0/1	66
AGIS_Os09g036360	LOC_Os09g30040	*OsAGP35P*	43.24	Yes	No	1/0/1/0	74
AGIS_Os10g003720	LOC_Os10g05150	*OsAGP36P*	35.21	Yes	No	0/1/1/1	71
AGIS_Os10g022660	LOC_Os10g28299	*OsAGP37P*	37.70	Yes	No	2/0/2/0	61
AGIS_Os11g035190	LOC_Os11g40850	*OsAGP38P*	36.62	Yes	No	1/1/1/1	71
AGIS_Os12g010080	LOC_Os12g11420	*OsAGP39P*	38.89	Yes	No	1/0/2/0	72
AGIS_Os12g010260	LOC_Os12g11610	*OsAGP40P*	42.65	Yes	No	1/1/0/1	68
AGIS_Os12g010270	LOC_Os12g11620	*OsAGP41P*	43.48	Yes	No	1/0/0/1	69
AGIS_Os12g010590	LOC_Os12g11980	*OsAGP42P*	42.65	Yes	No	1/1/0/1	68
AGIS_Os12g010600	LOC_Os12g11990	*OsAGP43P*	44.12	Yes	No	1/0/0/1	68

SP: signal peptide. GPI: GPI anchor addition sequence. AP/PA/SP/TP repeats: Ala–Pro, Pro–Ala, Ser–Pro, or Thr–Pro repeats. Yes indicates the existence of this structure, No indicates the absence of this structure.

## Data Availability

Data is contained within the article and Supplementary Materials.

## Supplementary Materials

The following supporting information can be downloaded at https://www.mdpi.com/article/10.3390/ijms26062616/s1.
