# Supplementary material for: Determining the Role of OsAGP6P in Anther Development Within the Arabinogalactan Peptide Family of Rice (Oryza sativa)
Source: Int J Mol Sci. 2025 Mar 14;26(6):2616. doi: 10.3390/ijms26062616 (PMC11941891; doi:10.3390/ijms26062616)
Supplement: Supplementary file 1 [file ijms-26-02616-s001.zip › Supplementary Materials/Supplementary Materials S1:Python Script.docx]

**Supplementary Method 1：Python Script**

import numpy as np

import pandas as pd

def read_fasta(filename):

sequences={}

seq_id=None

seq=None

with open(filename) as fa:

for line in fa.readlines():

line=line.strip()

if line.startswith('>'):

if seq_id:

sequences[seq_id]=seq

seq_id=line[1:]

seq=''

else:

seq+=line

if seq_id:

sequences[seq_id]=seq

sequences=pd.DataFrame({'seq':sequences})

return sequences

def AGP_select(sequences,len_min,len_max,past_min):

sequences=sequences.copy()

sequences['len']=sequences['seq'].map(len)

sequences['PAST']=sequences['seq'].map(

lambda x:sum([1 if s in 'PAST' else 0 for s in x ]))

sequences['PAST']=100*sequences['PAST']/sequences['len']

sequences_select=sequences[(sequences['len']>len_min)&(

sequences['len']<len_max)&(sequences['PAST']>=past_min)]

return sequences_select

def output_AGT_select(sequences,filename):

with open(filename,'w') as ofa:

for seq_id,seq in sequences.iterrows():

ofa.write('>'+seq_id+' LEN='+str(

seq['len'])+' PAST='+str(seq['PAST'])+'\n')

ofa.write(seq['seq']+'\n')

sequences=read_fasta('NIP-T2T.pep.fa')

select1=AGP_select(sequences,50,75,35)

output_AGT_select(select2,'AGT_50_75_PAST35.fa')
