# Supplementary material for: Determining the Role of OsAGP6P in Anther Development Within the Arabinogalactan Peptide Family of Rice (Oryza sativa)
Source: Int J Mol Sci. 2025 Mar 14;26(6):2616. doi: 10.3390/ijms26062616 (PMC11941891; doi:10.3390/ijms26062616)
Supplement: Supplementary file 1 [file ijms-26-02616-s001.zip › Supplementary Materials/Supplementary Materials Table S1 Primers and sequences in this study.docx]

**Table S2 Primers and sequences in this study**

| Primer name | Forward sequence (5’-3’) | Reverse sequence (5’-3’) |
| --- | --- | --- |
| *OsAGP6P*-pR | TATCCAGATCCAGTGGGATCCATGGCGCTCATCTCGAGAGC | TTGGTACCGAGCTCACCCGGGGAAGAGCTTGTAGGCGGAGGA |
| *OsAGP6P*-pRYJ | TTTGGTTTGCCCTTTTCCT | CTTGGTCCACGCTTGTTCC |
| *OsAGP6P*-GUS | TATGACCATGATTACGAATTCCCACTCTCACTGGCTAAGA | TGGCTGCAGGTCGACGGATCCCACAAGAACAGAAGCTCTCGA |
| *OsAGP6P*-GUS-Identify | ACCCCAGGCTTTACACTTTAT | TAGTTCCTTGGCAGTTCTTTT |
| Hyg | GAGCATATACGCCCGGAGTC | CAAGACCTGCCTGAAACCGA |
