# Supplementary material for: Determining the Role of OsAGP6P in Anther Development Within the Arabinogalactan Peptide Family of Rice (Oryza sativa)
Source: Int J Mol Sci. 2025 Mar 14;26(6):2616. doi: 10.3390/ijms26062616 (PMC11941891; doi:10.3390/ijms26062616)
Supplement: Supplementary file 1 [file ijms-26-02616-s001.zip › Supplementary Materials/Supplementary Materials Table S2 AG peptides identified in the rice genome.docx]

**Table S3 AG peptides identified in the rice genome**

| **Search criteria** | **No. of members** |
| --- | --- |
| Basic | 328 |
| Basic+ SP&GPI | 56 |
| Basic + AP peptides | 43 |
| Others | 13 |

Basic Criteria: PAST≥35%, 50<AA length<75. PAST: the proportion of Pro, Ala, Ser, and Thr. SP: Signal Peptide. GPI: GPI anchor addition sequence.
