# Supplementary material for: Determining the Role of OsAGP6P in Anther Development Within the Arabinogalactan Peptide Family of Rice (Oryza sativa)
Source: Int J Mol Sci. 2025 Mar 14;26(6):2616. doi: 10.3390/ijms26062616 (PMC11941891; doi:10.3390/ijms26062616)
Supplement: Supplementary file 1 [file ijms-26-02616-s001.zip › Supplementary Materials/Supplementary Materials Table S3 The protein backbones of rice AG peptides.docx]

**Table S4 The protein backbones of rice AG peptides.**

| **Gene name** | **Protein backbones** |
| --- | --- |
| *OsAGP1P* | MPARLREVITSFCCFLLLLLWCLGDGVCTGGRGRRRSSCTSGATSGHCRTDGPATAAAFCSASSASAGCCCRR |
| *OsAGP2P* | MARFSAAAVIAFAVVAAAALATVASAADAPAPAPTSGAVAAVSAPLSSFGVMGLGSVYLPLEYTVLRDRY |
| *OsAGP3P* | MASLKLRACVLLSTVFFFSGLMQLSMAQAGSPAAATATATTARVIDVKAVDQAVAYLLMLAALFVTYLAH |
| *OsAGP4P* | MATLKVYLLLFTAFFLSGLMQLSTATQTAPAPAAAPARVIDAKAIDQAIAYLLMFAALQSTYSKMEQIYDIS |
| *OsAGP5P* | MRAVGALLAAASCLPAPRAPTCPTTCCMLRLPRATVTHRLAQQRSSRCVRTSDVAAACRCSRQSHRSVA |
| *OsAGP6P* | MALISRASVLVMAIVAMLATLVNAQAPAPAPTSDGTSVDQGIAYVLMFVALALTYLIHPLDASSAYKLF |
| *OsAGP7P* | MAASTKALALLFAVLVALVAATAAVRVLEEEAVELGGLAPAPAPANAAGAVAPGAWAVAAVVSLLAFLAH |
| *OsAGP8P* | MAATLKLRILAAAATAAVVASSLVGVATATEAPAPGPSGASAGAAATNAAPAVALGTLAAAAVGYLFC |
| *OsAGP9P* | MAGQLKSKIVAVAVAAVVVVASSLVGTASAADAPAPAPTSGATATAAAAPAFAAVSVAAAALGGYLFC |
| *OsAGP10P* | MSTSSLHAFVVFFVMQVCLLVVMASSSSWTVQGRPLALESVPACCFYHPDCCQAAGADPAIADP |
| *OsAGP11P* | MARLQRRRLVLALLQLAVALWLAATSGCFCRQPPSPIAGVSSPPILPDGNRPPKSPPGPSDQIGTSSKTIS |
| *OsAGP12P* | MRLYSCAGGARAKSTAPAPRHRTRWPPHCSLLLVLVQAGAAGAAAMEDGGSRDSDHGVETSSPPKL |
| *OsAGP13P* | MAVAARAPLGVVAVVAVLVVGIFMPAAAQAPAPAPVSDGTSIDLGIAYILMLVALVLTYLIHPLDASSPYKLF |
| *OsAGP14P* | MTCSFLPPLLVGLLLSPPPRGADATRSPPARSLTLQRCAAPYRRPRTRGWAGRAYRSCSRRPSH |
| *OsAGP15P* | MASRGAVLVAVAAAVAVMFASVASAQVDGGVPPAPAPVTGAAAGGAASAALAVACSAVLSILVAGGIMH |
| *OsAGP16P* | MSMRKLIRLTVILALLVAAVAVSQCAAAEAAAAAGGMMRPRPPRRAAADWHVAAVSASPPANVTANLGADVLL |
| *OsAGP17P* | MGGGAVRKLLLLILALVATAAHAARAAPGTGANGDAVTRAARSLLSRASCCTHDGNTLTPNNPDYNNPKLNQTRP |
| *OsAGP18P* | MATTPRLPVVLLVLAAAALAAPAMADYAHPPLDPNQISVELAQFDRVVFSIYYEQPPFDRQSCRTAPVR |
| *OsAGP19P* | MARWHFLLAFAIRLCVPAPSELVVSLLGSTAVGRALAGHPDPFRVVHRSELAERRSSGSALTRSSPS |
| *OsAGP20P* | MARALVIAVILLAAIAVAPFAEASTVTTTSRVLLASEAPAESPAGPAEAPGPAEESSSASAPSPSAADA |
| *OsAGP21P* | MARALVLVVILLAAIAVAPFAEASTVSAASRVLLASEAPAESPAGPAPAPGPTEESSSTSAESPSADA |
| *OsAGP22P* | MARALVLVVILLAAIAVAPFAEASTVTGGSRVLLASDAPAESPAGPAAAPGPAEESSSESAPSPSAADA |
| *OsAGP23P* | MARIPFAAIVVAILSFAIAAAAQAPAPSPTSDGTSVDQGIAYLLMIVALVLTYLIHPLDASSAYKLF |
| *OsAGP24P* | MEMKKIACAVLVAASATVALAVEAPAPAPTSAAATSAAFPAVGAVIGASMLSFFAYYLQFKFQKVTTSNNILKQ |
| *OsAGP25P* | MAAAWTSLGLAAVAVVVVGIAMPASASAAAPAQPPAPAPSSDGVGFVFRNDME |
| *OsAGP26P* | MAALLAVVCLLRSSISCVATVDAATDNRHSCVVSAVAARPPIPALPASSREKTERGVSDLCESLDQA |
| *OsAGP27P* | MALPIAFLAVLAIAVKKATKAHTRTYPLEAVMLPATEQLLQPAPSPSPPPPPPQCDGQRLVGGDAAVEIDS |
| *OsAGP28P* | MSCRLGTMVALVLVGVVLAAILQEATVDAASSSSDSPAAKSGTGYLDYGNLKAKLPPPGVAVTKRPCIAKEKCRG |
| *OsAGP29P* | MGLLSTAAVLFSLAVAVAPPLVRLHQWYAAEFRGLNWAGAPPPLAALRRHPLRGLAPPPSSPGSPSSPQW |
| *OsAGP30P* | MAGLRMKALAVAAIAASLVASAAADHAPAPAPVSDAAPAFKEHEYRVLHGDTCMMLIVIYWPIV |
| *OsAGP31P* | MGSRKRGGGAVAMAVAMLLAAAAAAASASQPSSLEGFQPLSKIAVHKATVDLHGSAFVSATPALLGDQVDDE |
| *OsAGP32P* | MGFSHAIAILFIFLVASPPSPSHARMVPNDDTQHVPPTPAKGGAGRSRALWSAPSDGVGH |
| *OsAGP33P* | MTKMSFSSSALVLVLPSVVSGTTGSQVRAPALARAKGSEPAGSRTASWRRDQARTSSSWWCCST |
| *OsAGP34P* | MVVVVLLVAAASLHAADGAAAAPRRVLGADGGGGDQSESKVEHKSGCTNNDNTPPSGVCPPNAPGP |
| *OsAGP35P* | MPRALIALASAVLLLVAAVAPPLAAADDGGAGVPGEGKLESAGSAIKSAAANAFGVGSDIGGVPVNPSPGGANA |
| *OsAGP36P* | MPPSYTPTARLRRLVLFLAALSLLSPKPWPHPHGLGGRGLQSLGYIPRVRQGEQGPAVPRATLPPGPGLRR |
| *OsAGP37P* | MASSATSGRVLAIMLLMAIIAALMIINSPVAECRVAPDQVGVDPNGHCYFDPSSCRSPGAP |
| *OsAGP38P* | MNTTSFLLQILLLLAMVILMAMAMEPAAPETMARRSPGNVNANHDPSKQSGSAVHPLNTPCNYPGQAGCPH |
| *OsAGP39P* | MAGGWTSTASTGGGRQLVVPVLLLLVLTLLMVMASAAGRGANVNMVCVPRPSPEGCPIPPCGSPEYAPCPPP |
| *OsAGP40P* | MAAALRTTGGRRLAEVLVMMMFVLAAALLEAPAMASAARVLLQSGQLPTPYPTCNPGQYPCPSRTTVP |
| *OsAGP41P* | MAAALRATGGRRRLAALLVLMLFVMAAALQEAPVMASAARVLLQSGLLPTPYPTCNPGQYSCPPPTTVP |
| *OsAGP42P* | MAVALRTTGGRRLAAVLVMMLFVLAAALLEAPAMASAARVLLQSGQLPTPYPTCNPGQYPCPSRTTVP |
| *OsAGP43P* | MAAALRATGGRRLAAVLVLMLFVLAAALLEAPVMASAARVLLQSGLLPTPYPTCNPDQYSCPPPTTVP |

XXX, N-terminal signal peptides. AP, PA, SP, and TP repeats. XXX, GPI-anchor addition signal
