# Supplementary figures and images for: Determining the Role of OsAGP6P in Anther Development Within the Arabinogalactan Peptide Family of Rice (Oryza sativa)
Source: Int J Mol Sci. 2025 Mar 14;26(6):2616. doi: 10.3390/ijms26062616 (PMC11941891; doi:10.3390/ijms26062616)

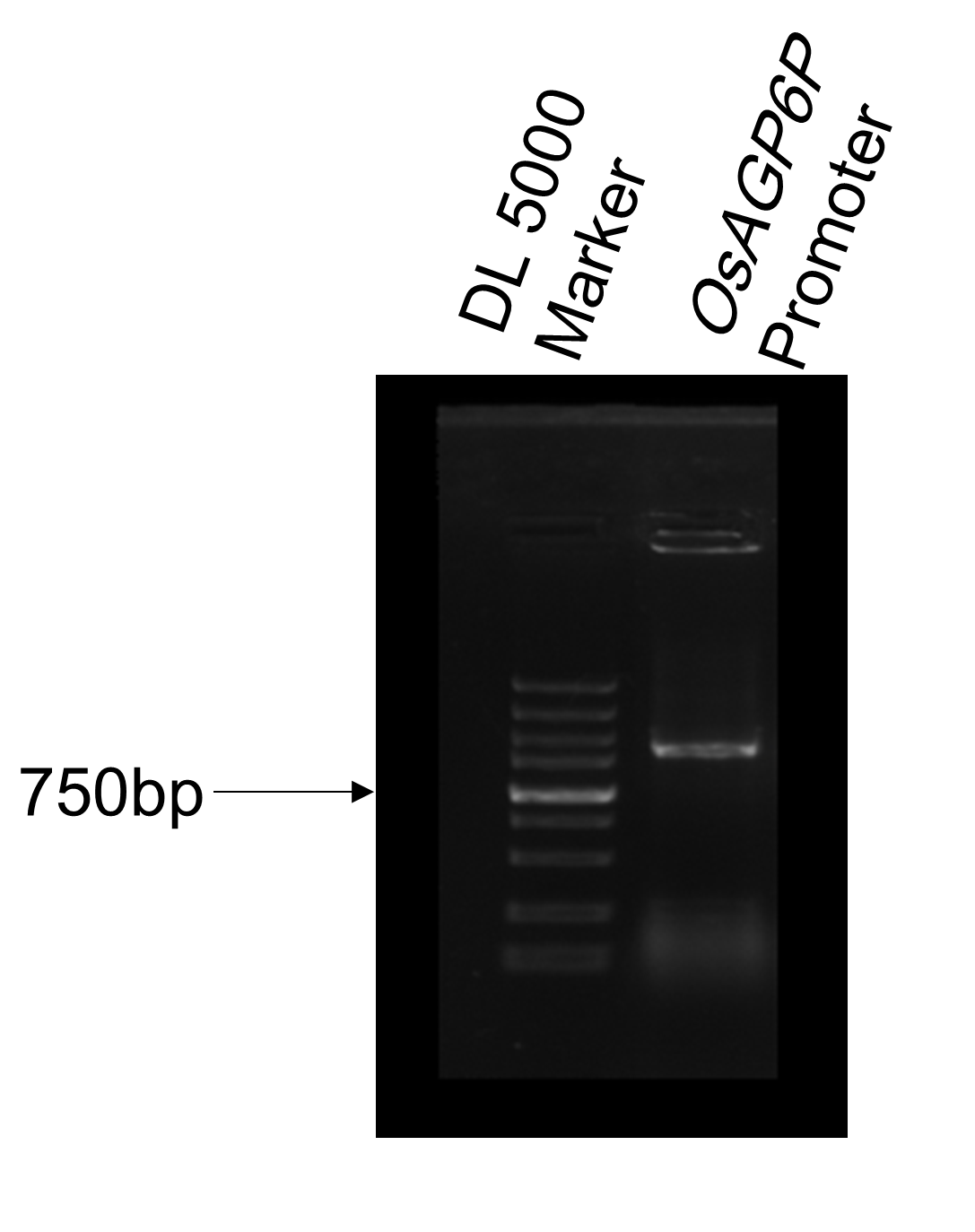

Supplement: Supplementary file 1 [file ijms-26-02616-s001.zip › Supplementary Materials/Supplementary Materials Figure S1 Promoter amplification electropherograms of OsAGP6P.png]

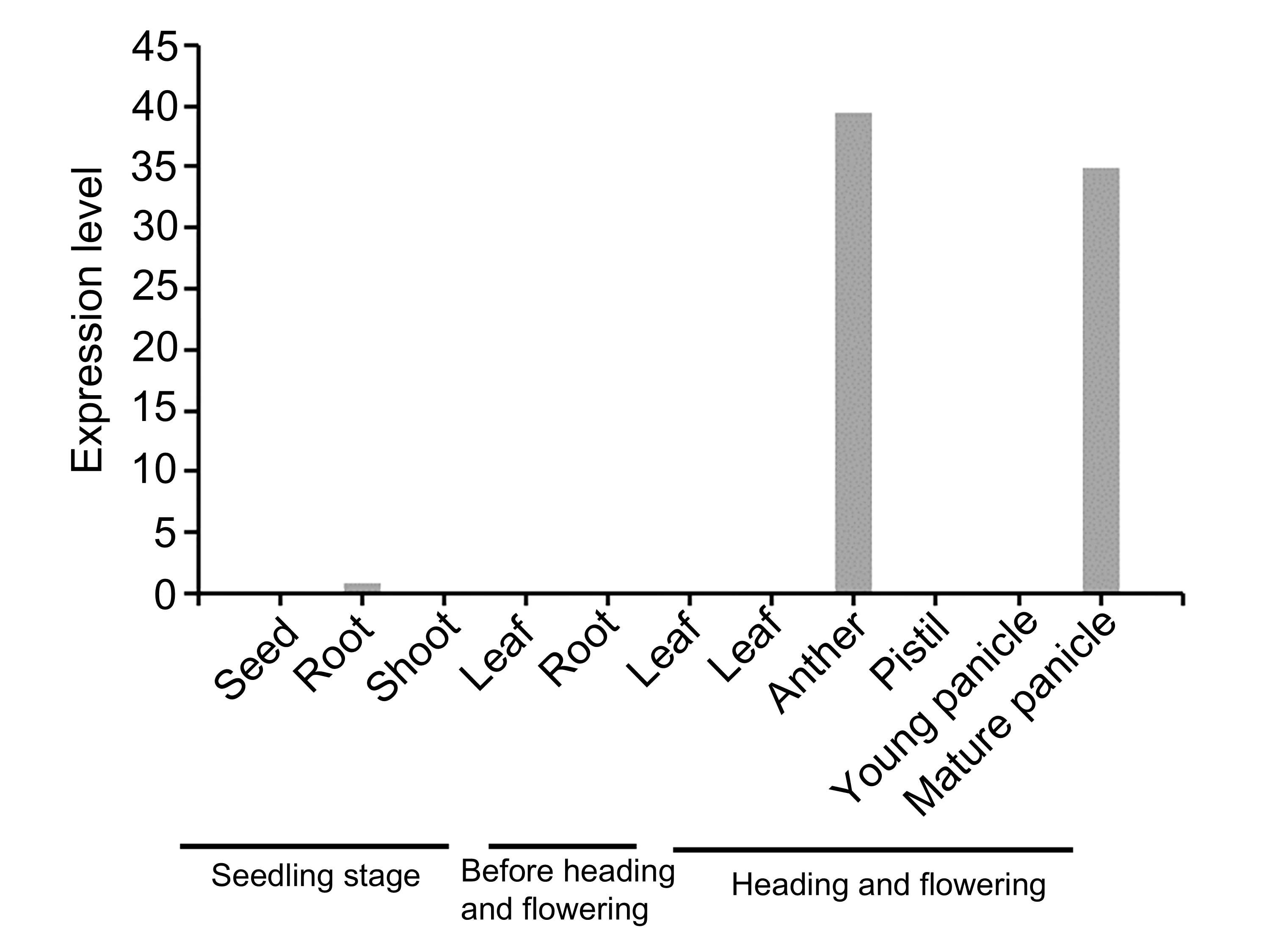

Supplement: Supplementary file 1 [file ijms-26-02616-s001.zip › Supplementary Materials/Supplementary Materials Figure S2 Expression levels of OsAGP6P in rice tissues.png]
